# Phosphatidylinositol 3-phosphate metabolism impacts cellular α-synuclein localization in *Saccharomyces cerevisiae*

**DOI:** 10.1016/j.jbc.2025.110666

**Published:** 2025-09-01

**Authors:** Timo Löser, Fazilet Bekbulat, Christian Behl, Jana Schepers

**Affiliations:** The Autophagy Lab, Institute of Pathobiochemistry, University Medical Centre of the Johannes Gutenberg University, Mainz, Germany

**Keywords:** Parkinson disease, α-Synuclein, yeast, PI3 kinase, proteostasis

## Abstract

Alpha-synuclein (αSyn), a hallmark protein of synucleinopathies such as Parkinson’s disease (PD), is likely to be involved in neuronal membrane trafficking and synaptic vesicle dynamics at axon terminals. Its specific binding to anionic phospholipids, such as phosphatidylinositol phosphates that are essential for intracellular signaling and membrane trafficking, suggests an involvement in vesicular transport processes. In *Saccharomyces cerevisiae*, a model organism for cell biological PD research, human αSyn localizes to the plasma membrane *via* the secretory machinery. Employing this yeast model, we investigated the impact of αSyn on cellular quality control mechanisms. Additionally, we focused on the effect of αSyn expression in yeast mutants impaired in specific phospholipid biosynthesis and transport pathways, including endovacuolar trafficking and autophagy. In the deletion strains *vps34Δ* and *vps15Δ*, lacking phosphatidylinositol 3-phosphate (PI3P) biosynthesis, αSyn mislocalizes in the cytosol, and significantly reduces cell viability. In these strains, αSyn species containing an intact lipid-binding N terminus also form large perivacuolar, lipid-rich accumulations. In wild type cells, αSyn expression alters the morphology of PI3P-rich membrane structures and upregulates transcription of *SEC4*, which encodes a key regulator of the late secretory pathway. Moreover, αSyn colocalizes with overexpressed Sec4 at the emerging cell bud. Our findings demonstrate that PI3P is critical for the targeting of αSyn to the yeast plasma membrane *via* the secretory pathway, revealing a potential entry point into this complex machinery. Understanding the relationship between αSyn and vesicular trafficking in this system will enhance our knowledge of αSyn-trafficking in mammalian cells and, eventually, in PD, offering new research avenues.

Parkinson’s disease (PD) is a progressive neurodegenerative disorder primarily characterised by the loss of dopaminergic neurons in the *substantia nigra*. This leads to motor symptoms, such as resting tremors, slowness of movements (bradykinesia), and rigidity (1, 2). On the molecular level, PD pathogenesis is closely linked to the aggregation of dysfunctional alpha-synuclein (αSyn), which forms Lewy bodies and Lewy neurites, the pathological hallmarks of synucleinopathies, such as PD and Lewy body dementia (3, 4, 5).

Physiologically, αSyn is predominantly expressed in neurons and plays a role in various vesicular transport and membrane fusion processes in the neuronal soma and axon terminals. Proposed functions of αSyn include the regulation of synaptic vesicle transport and neurotransmitter release at the presynapse, as well as involvement in endoplasmic reticulum–Golgi trafficking, intra-Golgi transport, and the endosomal shuttle network (6, 7, 8, 9, 10, 11, 12). For these functions, αSyn preferably binds to anionic membrane phospholipids (PLs) *via* six N-terminal KTKEGV domains (13, 14). Membrane fusion is facilitated through the acidic C-terminal region that mediates protein–protein interactions with, for example, t-SNARE proteins and Rab GTPases (15, 16).

PD pathology suggests a disruption of αSyn-related membrane trafficking processes as various vesicular structures, including autophagosomes, endosomes, and whole organelles, have been identified in Lewy body–like structures (17, 18, 19). The baker’s yeast *Saccharomyces cerevisiae* is frequently used as a model organism in PD research to further explore the relationship between αSyn’s PL interactions, its cellular localization, and αSyn cytotoxicity. When expressed in yeast, αSyn localizes to budding sites at the plasma membrane (PM) and accumulates there in a dose-dependent manner, which leads to severe growth inhibition (20, 21). In addition to WT αSyn, studies often include two mutated αSyn isoforms, A30P and A53T, which are associated with familial forms of PD (22, 23). Unlike WT and A53T αSyn that target the PM, A30P localizes in the cytoplasm and has a significantly lower impact on growth rate and cell viability (20).

Previous research has shown that WT and A53T αSyn require an intact secretory pathway for correct PM localization. The absence of key secretory proteins, such as Sec4, Sec9, and Sec14, leads to cytoplasmic accumulation of αSyn (21). Similar to observations in mammalian cells, human αSyn also colocalizes with yeast Rab GTPases, including Ypt6, Ypt31, and Ypt32, and high αSyn levels disrupt endosomal trafficking (24, 25). Furthermore, genetic deletions affecting lipid metabolism and vesicle-mediated transport processes induce synthetic lethality upon αSyn expression (24, 26). High αSyn levels in wild type yeast strains also impair the ubiquitin-proteasome system and increase expression of the autophagy-reporter protein Atg8 but not autophagic activity itself (20, 27, 28, 29). However, it was shown that aged cells demonstrate an increase in autophagic activity upon αSyn expression (30).

In this study, we investigated the dose-dependent effects of human αSyn expression on cell viability and cellular quality control pathways, particularly on autophagy and the ubiquitin-proteasome system. Our yeast screen of autophagy-related (ATG) gene deletion strains links αSyn mislocalization and toxicity to defective biosynthesis of phosphatidylinositol 3-phosphate (PI3P), an anionic PL involved in endosomal trafficking and early stages of secretion (31, 32). This is interesting, since in mammalian cells, αSyn localizes to pools of highly anionic phosphatidylinositol phosphates (PIPs) at the PM (33, 34). Our results demonstrate that, among all known yeast phosphatidylinositol (PI) kinases, only the PI3 kinase (PI3K) Vps34, its activator Vps15, and the PIKfyve ortholog Fab1 significantly influence the localization of the membrane-binding WT and A53T αSyn isoforms in wild type yeast.

For the first time, we show that human αSyn upregulates the gene expression of *SEC4*, involved in late stages of secretion, colocalizes with Sec4 at emerging bud sites, and induces the formation of multiple small PI3P-rich structures in the cytoplasm. Taken together, our findings also suggest that αSyn is able to enter the highly conserved secretory pathway of *S. cerevisiae* through interactions with PI3P, which appears similar to its putative functions at presynaptic axon terminals (35).

## Results

### Impact of αSyn expression on proteostasis and cellular stress pathways

Expression of human αSyn from a 2 μ plasmid in wild type yeast strains is known to reduce cell growth and induce expression of the autophagy reporter protein Atg8 (27). Therefore, we investigated whether an *atg1Δ* deletion strain, deficient for macroautophagy, is more sensitive to αSyn expression. To this end, we expressed C terminally GFP-tagged WT, A53T, and A30P αSyn in both, the WT and the *atg1Δ* strains (Fig. S1). To assess dose-dependent effects of αSyn-GFP expression, we used one-copy (*CEN/ARS*) and high-copy (2 μ) expression constructs.

**Figure 1:**
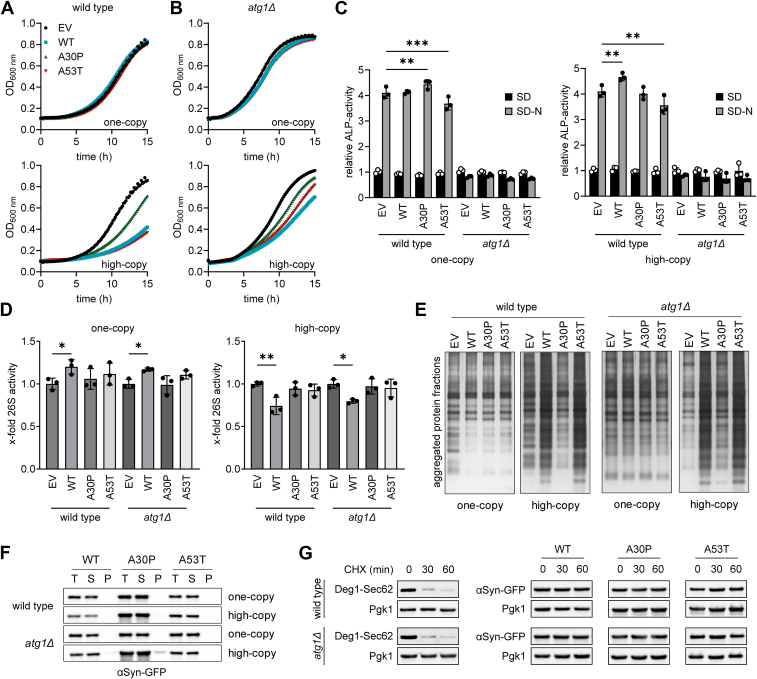
**Dose-dependent influence of human αSyn-GFP on growth and proteostasis in *Saccharomyces cerevisiae.****A*–*F*, experiments were conducted with strains expressing αSyn-GFP from one-copy (CEN/ARS) or high-copy (2 μ) plasmids. *A*, growth curves of wild type and (*B*) *atg1Δ* strains expressing WT, A30P, or A53T αSyn-GFP or the empty vector (EV) control. Growth inhibition can only detected when αSyn-GFP is expressed from a high-copy plasmid. *Top*: Expression from one-copy constructs, *bottom*: expression from high-copy constructs. N = 3. *C*, Pho8Δ60 assay in wild type and *atg1Δ* strains prior to (0 h) and after 16 h of nitrogen starvation, expressing the one-copy (*left*) or high-copy (*right*) WT, A30P, A53T αSyn-GFP constructs or the EV. Autophagic activity increases upon N-depletion and is not significantly affected by αSyn-GFP expression. Pho8 activity was normalized to the wild type EV control before N-starvation (0 h). *D*, relative proteasome activity in wild type and *atg1Δ* strains expressing one-copy (*left*) or high-copy (*right*) WT, A30P, or A53T αSyn-GFP constructs. One-copy expression of WT αSyn-GFP slightly increases proteasomal activity, whereas high-copy expression of WT αSyn-GFP results in decreased proteasome activity. Values are normalized to the wild type EV control. *C*–*D*, values represent mean ± standard deviation; N = 3. ∗*p* < 0.05; ∗∗*p* < 0.01; and ∗∗∗*p* < 0.001. *E*, silver stain of aggregated protein fractions in polyacrylamide gels. Aggregated fractions were extracted from wild type and *atg1Δ* strains expressing WT, A30P, or A53T αSyn-GFP or the EV control from the one-copy or the high-copy constructs and separated *via* SDS-PAGE. Protein aggregation is elevated in wild type and *atg1Δ* strains, when expressing WT and A53T αSyn-GFP from high-copy plasmids. N = 3. *F*, Western blot analysis of total lysate (T) soluble proteins (S) and aggregated fractions (P) from wild type and *atg1Δ*, expressing WT, A30P, or A53T αSyn-GFP. αSyn-GFP is almost exclusively found in the soluble protein fractions. Primary antibody: α-GFP (mouse) 1:2000; N = 3. *G*, immunodetection of unstable degradation reporter Deg1-Sec62 and αSyn-GFP (WT, A30P or A53T) 0, 30, and 60 min after cycloheximide (CHX) treatment. Pgk1 was used as loading control. α-Syn-GFP is not degraded within 60 min after CHX treatment. Primary antibodies: α-mouse-horseradish peroxidase (goat), α-GFP (mouse), and α-Pgk1 (mouse); N = 3. αSyn, alpha-synuclein.

In accordance with previous findings (20), expression of any αSyn-GFP variant from the one-copy construct has no growth effects on the wild type, while expression from the 2 μ construct results in a significant growth decline, which is less severe with the A30P isoform (Fig. 1*A*). Unexpectedly, however, deleting *ATG1* does not exacerbate growth inhibition by αSyn-GFP expression. The *atg1Δ* strain shows very similar growth reduction to the wild type strain upon high-copy αSyn-GFP expression (Fig. 1*B*).

Next, we assessed whether αSyn-GFP expression affects autophagy in general by performing a Pho8Δ60 assay in both the wild type and, as a negative control, the *atg1Δ* strains (36). In the wild type, autophagic activity increases approximately 4-fold after 16 h of nitrogen starvation. Interestingly, expression of αSyn-GFP from both, one-copy and high-copy constructs, does not affect autophagic activity in nonstarved cells and shows only slight additional effects after 16 h of nitrogen starvation (Fig. 1*C*). As anticipated, in the *atg1Δ* controls, Pho8 activity does not increase upon nitrogen depletion. These data are consistent with earlier studies that have demonstrated that, although Atg8 expression is induced by αSyn expression, autophagic activity remains unchanged in exponentially growing cells (27, 30).

A recent study has shown that expressing WT αSyn-GFP from a high-copy plasmid reduces chymotrypsin-like proteasome activity in wild type yeast (37). As protein degradation in the *atg1Δ* strain predominantly relies on the ubiquitin proteasome system, we assessed the activity of the 26S proteasome upon expression of the one-copy and high-copy WT, A53T, and A30P αSyn-GFP constructs. Sample treatment with 100 μM of the proteasome inhibitor MG132 confirmed proteasome specificity of this assay (Fig. S2). In the empty vector (EV) control, both the wild type and *atg1Δ* exhibit similar proteasome activity (Fig. 1*D*). Expression of WT αSyn-GFP from a one-copy construct increases 26S proteasome activity significantly by 20% in both strains, whereas A30P and A53T αSyn-GFP cause no increase. In contrast, expression of WT αSyn-GFP from the high-copy constructs leads to a significant decrease of proteasomal activity by 20% in the wild type and in *atg1Δ*. This has previously been published for wild type strains but not for the autophagy-deficient *atg1Δ* strain (37). Similar to the one-copy expression systems, we could not detect any significant changes in proteasomal activity in the high-copy A30P and A53T expressing strains. Our findings suggest that expression of WT αSyn-GFP affects proteasomal activity in a dose-dependent manner, which occurs independently of a functional autophagy pathway.

Next, we investigated whether decreased proteasomal activity influences protein aggregation, especially in the autophagy-deficient *atg1Δ* strain, using an in-gel protein aggregation assay. Strikingly, high-copy expression of both, WT and A53T αSyn-GFP, results in a significant increase of aggregated proteins in both the wild type and the *atg1Δ* strain (Fig. 1*E*). Western blot analysis of these fractions revealed that αSyn-GFP is absent or only slightly present in the insoluble fractions (Fig. 1*F*). These findings suggest that the expression of WT and A53T αSyn-GFP promotes the aggregation of yeast proteins other than αSyn-GFP.

Lastly, we addressed whether the cell is capable of degrading αSyn-GFP by performing a cycloheximide (CHX) chase assay. As expected, most of the unstable reporter protein Deg1-Sec62 is degraded within 30 min (Fig. 1*G*) (38). In contrast, no significant degradation of any αSyn-GFP species is detected after 1 h of CHX treatment. Since we found that αSyn-GFP expression from one-copy constructs does not significantly affect protein degradation pathways in yeast, it appears that αSyn-GFP is either inherently stable or otherwise protected from degradation.

Taken together, our data show that autophagy dysfunction in the *atg1Δ* strain does not worsen αSyn-GFP–induced growth defects, nor does the expression of αSyn-GFP elevate overall autophagic activity, suggesting that αSyn-GFP sensitivity occurs independently of the (macro-)autophagy pathway. Moreover, high-copy expression of both WT and A53T αSyn-GFP enhances protein aggregation in yeast, yet only WT αSyn-GFP reduces proteasomal activity. This suggests that WT and A53T αSyn-GFP may block aggregate clearance through a separate mechanism, possibly linked to their membrane-binding properties, rather than proteasomal impairment alone.

### Localization of αSyn-GFP and growth inhibition is linked to phosphatidyl-inositol phosphate metabolism

**Figure 2:**
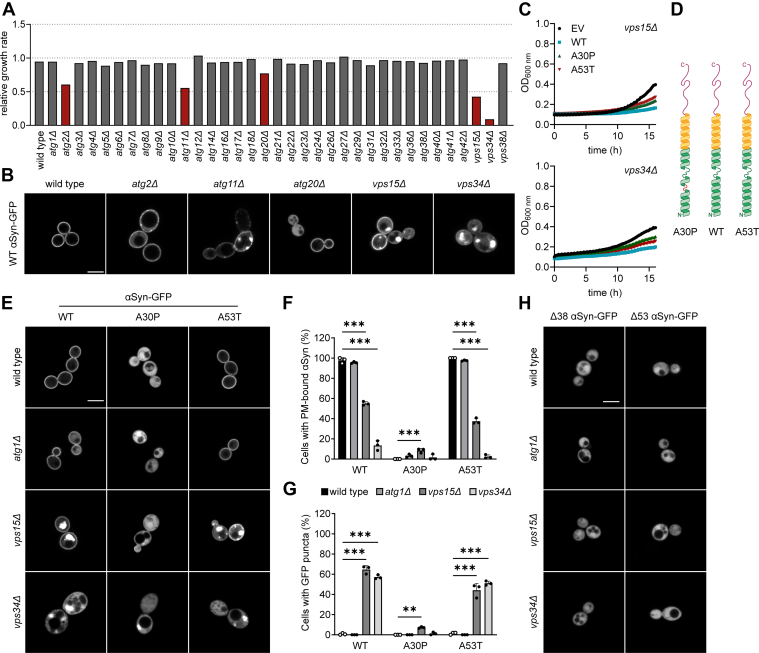
**Effect of *ATG* gene deletion on αSyn localization and toxicity.** All experiments were conducted with cells expressing αSyn from one-copy plasmids. *A*, relative growth rate screening of *ATG* gene deletion mutants expressing human WT αSyn-GFP. The growth of exponentially growing cells was measured and normalized to the EV control. Only the *atg2Δ*, *atg11Δ*, *atg20Δ*, *vps15Δ,* and *vps34Δ* strains exhibit growth inhibition by WT αSyn-GFP expression. N = 1. *B*, microscopic imaging of WT αSyn-GFP–expressing strains that showed a growth reduction of > 20% in (*A*). WT αSyn-GFP is localized (at least partially) in the cytoplasm of *atg2Δ*, *atg11Δ*, *atg20Δ*, *vps15Δ,* and *vps34Δ* strains. The scale bar represents 5 μm. *C*, growth curves of *vps15Δ* and *vps34Δ* expressing human αSyn-GFP compared to the EV control. Expression of αSyn-GFP from one-copy plasmids inhibits growth of *vps15Δ* and *vps34Δ* strains. *D*, simplified depiction of αSyn helicality in the presence of lipids. The A30P mutation causes a disruption of least one complete turn at the N-terminal helix, which inhibits membrane binding. *E*, fluorescent microscopy of WT, A30P, A53T αSyn-GFP in wild type, *atg1Δ*, *vps15Δ,* and *vps34Δ* strains. WT and A53T αSyn-GFP is mislocalized into the cytoplasm in *vps15Δ* and *vps34Δ* strains and forms large accumulations. The scale bar represents 5 μm. *F*, quantification of cells with membrane-bound αSyn and (*G*) intracellular GFP puncta. *F*–*G*, N = 3, with at least 100 cells per N. Values represent mean + standard deviation. ∗∗*p* < 0.01 and ∗∗∗*p* < 0.001. *H*, microscopic analysis of the wild type, *atg1Δ*, *vps15Δ,* and *vps34Δ* expressing the αSyn-GFP truncations Δ38 αSyn-GFP and Δ53 αSyn GFP. N terminally truncated αSyn-GFP does not accumulate as GFP-positive puncta in the cytoplasm. The scale bar represents 5 μm. αSyn, alpha-synuclein; ATG,autophagy-related; EV, empty vector.

Our data show that the autophagy deficiency in *atg1Δ* strains does not exacerbate growth inhibition caused by αSyn-GFP expression. However, autophagy is a complex machinery that is also dependent on lipid metabolism and an interplay of vesicular and intracellular trafficking pathways (39, 40, 41). Interestingly, deletion strains impaired in lipid metabolism and vesicle transport show increased synthetic lethality when expressing αSyn-GFP (26).Therefore, we aimed to investigate the effects of αSyn-GFP expression on the growth rate of several ATG deletion strains (Fig. 2).

To this end, we assessed cellular growth of the selected strains expressing WT αSyn-GFP from one-copy *CEN/ARS* constructs and determined their maximum growth rate normalized to each strain’s EV control. As expected, and similarly to the *atg1Δ* strain, most of the selected deletion strains do not show severe growth inhibition (Fig. 2*A*). However, exceptions are the deletion strains *atg2Δ* and *atg11Δ*, which show a growth reduction of approximately 40%, and the *atg20Δ* strain with a growth reduction of approximately 20%. Strikingly, the loss of the PI3K core units, Vps15 and Vps34, result in the strongest growth reduction of approximately 60% and 80%, respectively. To further investigate the affected deletion strains, we examined αSyn-GFP localization within the cells. In the wild type, αSyn-GFP localizes to the PM, as previously reported (20). In contrast, each of the deletion mutants shows, at least partially, diffuse GFP signals throughout the cytoplasm. For example, in the *atg20Δ* strain, only approximately half of the cells show PM binding of αSyn-GFP. Strikingly, large GFP-positive puncta can be found in the *atg11Δ,* and most prominently in the *vps15Δ,* and *vps34Δ* strains (Fig. 2*B*). In wild type strains, similar accumulations are only found when αSyn is expressed from at least two stable genomic copies (42). Since there are no observable differences in growth rate and αSyn-GFP localization in the *atg1Δ* strain, the observed growth inhibition in the *atg2Δ*, *atg11Δ*, *atg20Δ*, *vps15Δ,* and *vps34Δ* strains cannot be attributed simply to an impaired autophagic machinery. Notably, all these proteins are involved in the metabolism and intracellular trafficking of PIPs such as PI3P (43, 44, 45, 46, 47).

As Vps15 and Vps34 are two core components of the PI3P-synthesizing PI3K complex and exhibit the most severe growth inhibition upon WT αSyn-GFP expression, we compared cellular growth upon expression of the three αSyn-GFP isoforms WT, A30P, and A53T in the *vps15Δ*, and *vps34Δ* strains. Growth analysis shows that, in *vps15Δ* and *vps34Δ*, the expression of each αSyn variant from a single-copy plasmid suffices to further impair cellular growth (Fig. 2*C*), with WT αSyn-GFP having the most severe impact. Next, we also investigated WT, A30P, and A53T αSyn-GFP localization in both strains and compared it to the wild type and the *atg1Δ* mutant. The A30P mutation located in the N-terminal domain of αSyn partially disrupts helix formation in the presence of PLs and, thus, inhibits lipid binding (Fig. 2*D*) (48, 49). We found that both deletion strains have significantly fewer cells displaying membrane localization of WT and A53T αSyn-GFP compared with the wild type and the *atg1Δ* strain. The *vps34Δ* strain shows the most significant decrease of αSyn-GFP PM localization, with a reduction of 86% and 98% of WT and A53T αSyn-GFP, respectively. In the *vps15Δ* strain, only 45% of the cells show PM localization when WT αSyn-GFP is expressed and only 37% of the cells show PM localization of A53T αSyn-GFP (Fig. 2, *E* and *F*). We did not observe any detectable changes in A30P αSyn-GFP localization in either strain. Consistent with these findings, the cytoplasmic GFP accumulations in *vps15Δ* and *vps34Δ* are more abundant in strains expressing lipid-binding αSyn-GFP variants, with GFP puncta observed in 65% and 44% of WT αSyn-GFP-expressing cells, and 57% and 51% in cells expressing A53T αSyn-GFP, respectively (Fig. 2*G*). Furthermore, these puncta stain positive for FM4-64 but not for thioflavin T, suggesting the presence of membrane lipids accumulating with nonamyloid αSyn-GFP species (Figs. S3 and S4) (50). Cells expressing the A30P αSyn-GFP variant show significantly less GFP accumulations. This implies that lipid-binding αSyn isoforms are more prone to accumulate in the PI3K-mutant strains. To further confirm this, we examined the cellular localization of two GFP-tagged αSyn truncations, a Δ38 αSyn-GFP and a Δ53 αSyn-GFP, lacking three or four PL-binding KTKEGV motifs, respectively. When we express these truncations in the wild type, *atg1Δ*, and the PI3K mutant strains *vps15Δ* and *vps34Δ*, αSyn-GFP fails to bind to the PM in any of the strains and we cannot detect any cellular accumulations, even in v*ps15Δ* and *vps34Δ* (Fig. 2*H*).

Overall, our data indicate that, while the autophagy-deficient *atg1Δ* strain is not more sensitive to αSyn-GFP expression than the wild type, deletions in ATG genes involved in phosphoinositide metabolism and vesicular trafficking, especially *vps15Δ* and *vps34Δ*, lead to severe growth defects and promote cytoplasmic αSyn-GFP accumulations. Notably, the membrane-binding WT and A53T αSyn-GFP variants are more prone to accumulation than the A30P isoform or truncations lacking lipid-binding KTKEGV motifs, implying a critical role of phosphoinositide binding for “correct” αSyn-GFP localization. In order to understand the discrepancy between membrane binding in *vps34Δ* and *vps15Δ*, it is important to note that although Vps15 is an activator for Vps34, there are still small amounts of PI3P generated in the *vps15Δ* strains (51).

### Localization of αSyn-GFP depends on the catalytic function of PI3K

**Figure 3:**
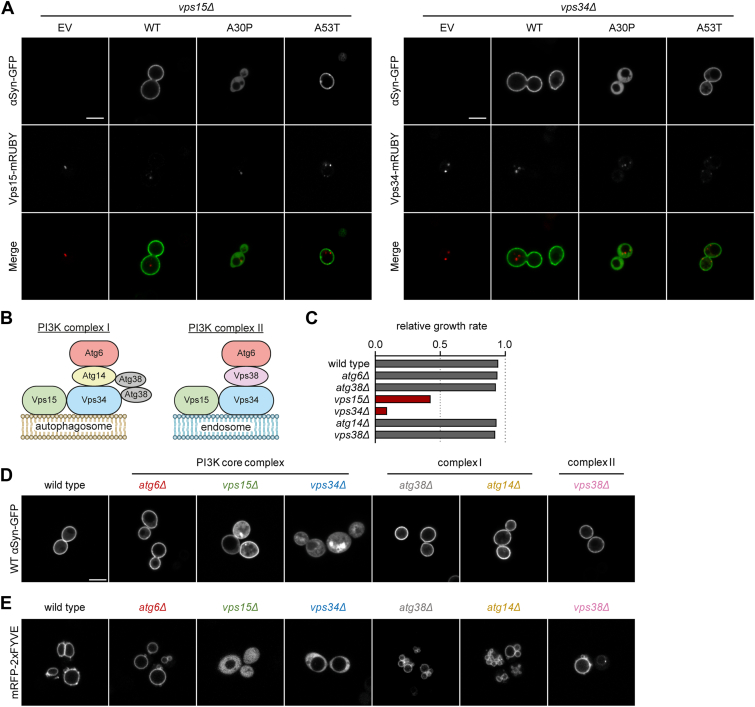
**PM localization of αSyn-GFP requires catalytically active PI3K complex II.** All analyses were performed with cells expressing αSyn-GFP from one-copy plasmids. *A*, fluorescence microscopy imaging of rescued *vps15Δ* and *vps34Δ* deletion strains expressing αSyn-GFP isoforms WT, A30P, or A53T or the EV control. Vps15 and Vps34 were localized by a C-terminal fluorescent mRUBY2 epitope. Reintroduction of either protein into the respective deletion mutant rescues αSyn-GFP localization. The scale bar represents 5 μm. *B*, simplified structure of the PI3K complexes I and II. *C*, relative growth rate of deletion strains lacking single PI3K components from Figure 2*A*. Exponential growth normalized to the EV control. *D*–*E*, microscopic localization of WT αSyn-GFP and PI3P in single deletion strains *atg6Δ*, *vps15Δ*, *vps34Δ*, *atg38Δ*, *atg14Δ*, and *vps38Δ*. PI3P was detected with an mRFP-2xFYVE reporter protein, expressed from a one-copy plasmid. Only the absence of Vps15 and Vps34 causes a disruption in PI3P biosynthesis and demonstrates mislocalized WT αSyn-GFP. The scale bar represents 5 μm. αSyn, alpha-synuclein; EV, empty vector; PI3K, PI3 kinase; PI3P, phosphatidylinositol 3-phosphate; PM,plasma membrane.

Whole gene deletions in the densely packed genome of *S. cerevisiae* are often associated with transcription defects for adjacent genes (52, 53, 54). To determine whether this “neighboring gene interference” is responsible for the observed mislocalization and accumulation of αSyn-GFP, we introduced one-copy plasmids carrying the *VPS15* and *VPS34* genes into the respective deletion mutants. We coexpressed the PI3K proteins with each of the αSyn-GFP isoforms (WT, A30P, or A53T) and examined αSyn-GFP localization. The reintroduction of either gene successfully restores the PM localization of WT and A53T αSyn-GFP in both strains (Fig. 3*A*). Furthermore, the cytoplasmic GFP accumulations are absent in both rescue strains, implying that a catalytically active PI3K is sufficient for PM localization.

In addition to the catalytic components Vps15 and Vps34, the yeast PI3K complex contains further structural components, which determine its cellular localization. PI3K complex I, responsible for PI3P generation at the preautophagosomal structure contains Atg6 (55), as well as the complex I specific proteins Atg14 and Atg38 (55, 56). Complex II, which synthesises endosomal PI3P, also contains Atg6 and the complex II–specific Vps38 (57) (Fig. 3*B*). Therefore, we further examined the localization of αSyn species in strains deficient for these proteins. In contrast to the *vps15Δ* and *vps34Δ* deletion strains, the *atg6Δ*, *atg14Δ*, *atg38Δ,* and *vps38Δ* deletion strains show no significant growth inhibition upon αSyn-GFP expression (Fig. 3*C*). Examining cellular localization reveals that αSyn-GFP correctly localizes to the PM in these deletion strains, with puncta formation occurring only in the *vps15Δ* and *vps34Δ* strains (Fig. 3*D*). To connect αSyn-GFP localization to PI3P, we next marked cellular PI3P, using mRFP-2xFYVE as a reporter protein (58) (Fig. 3*E*). It is evident, that the absence of PI3K components other than Vps15 and Vps34 does not disrupt PI3P biosynthesis overall. Furthermore, small punctate structures near the vacuoles indicate endosomal PI3P generation in all strains, including the complex II single deletion mutants (59).

**Figure 4:**
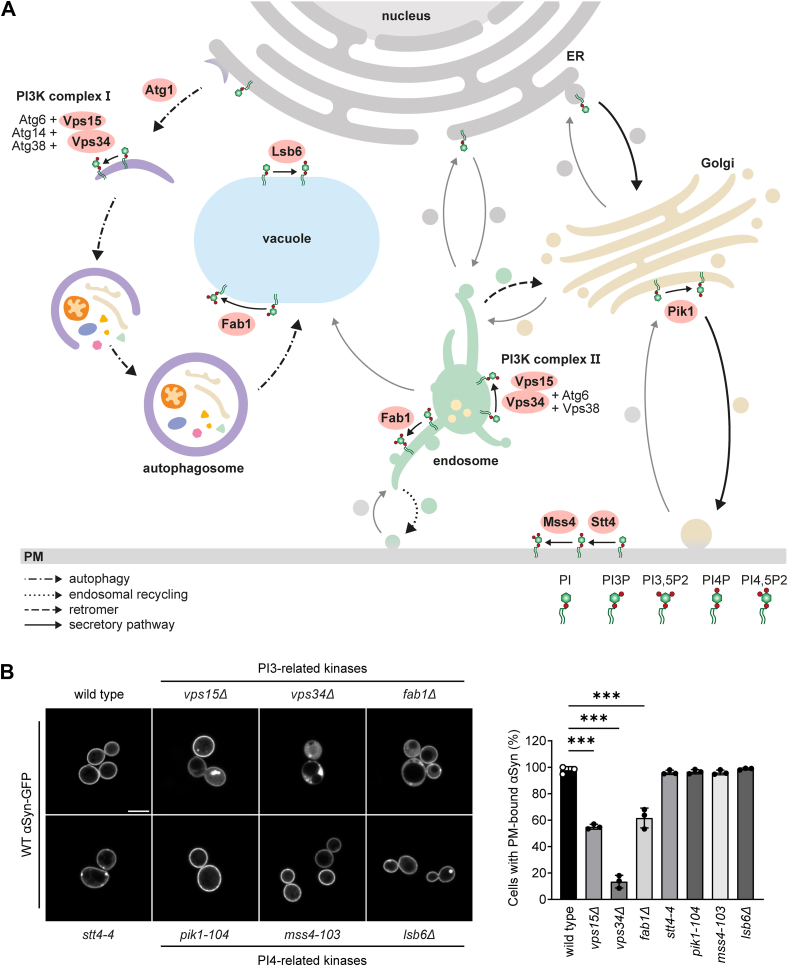
**PI3-related PIP species affect PM localization of αSyn-GFP.***A*, schematic overview of the PIP biosynthesis and relevant trafficking pathways in yeast cells. PI3P is generated by both, the PI3K complex I and II at phagophores and endosomes, respectively. PI(3,5)P2 is generated by Fab1 at vacuoles and endosomal structures. PI4P is generated by the three different kinases Lsb6, Pik1, and Stt4 at the vacuole, the late Golgi and the PM, respectively. Mss4 synthesises PI(4,5)P2 from PI4P at the PM. The most relevant pathways for this study, involving these PIP kinases, include the autophagy machinery, endosomal recycling, retromer trafficking and the secretory machinery. *B*, localization and quantification of PM-bound WT αSyn-GFP in known PI-related kinase–deficient mutants. Deletion of PI3-related kinases Vps15, Vps34, and Fab1 results in cytoplasmic WT αSyn-GFP in at least 40% of the analysed cells. N = 3, with at least 100 cells per N. ∗∗∗*p* < 0.001. αSyn, alpha-synuclein; PI3K, PI3 kinase; PI3P, phosphatidylinositol 3-phosphate; PIP, phosphatidylinositol phosphate; PM, plasma membrane.

To determine whether a deficiency of other PIP kinases, and thereby other PIP species, can affect αSyn-GFP localization, we examined WT αSyn-GFP localization in *fab1Δ*, *pik1-104*, *mss4-103*, *lsb6Δ, and stt4-4* strains. Fab1 synthesises PI(3,5)P2 from PI3P; Lsb6, Pik1, and Stt4 catalyze the phosphorylation of PI to PI4P; and Mss4 converts PI4P to PI4,5P2. A graphical summary of the yeast PIP biosynthesis as well as the major pathways PIPs are involved in is shown in Figure 4*A*. It is important to note that since *pik1-104*, *mss4-103*, *and stt4-4* are conditional alleles, the levels of PI4-related species are significantly reduced but not depleted at 30 °C (60, 61, 62). Notably, only the loss of PI3 related kinases Vps15, Vps34, and Fab1 results in partial mislocalization of lipid-binding WT and A53T αSyn-GFP to the cytoplasm, with cytoplasmic accumulations exclusively found in the PI3K-deficient strains *vps15Δ* and *vps34Δ* (Figs. 4*B* and S5). Similarly, we observed small PM-localized WT αSyn-GFP accumulations in PI4K-insufficient strains *stt4-4* and *lsb6Δ*. Both Stt4 and Lsb6 synthesize PI4P from PI at the PM (63, 64). Lsb6 has been found at the yeast vacuole as well (63). As total PI4P is only reduced by approximately 30% at 30 °C growth temperature in the *stt4-4* strain, we also looked for αSyn-GFP localization in wild type cells incubated for 5 h with 3 μM Wortmannin. At this concentration, Wortmannin completely inhibits Stt4 and only reduces Vps34 activity by 50%. Similar to what we observed in the PI4K mutant strains, these strains exhibited PM-localized WT αSyn-GFP accumulations (Fig. S6). Growth analysis revealed that the expression of WT αSyn-GFP specifically impairs cell growth only in the *stt4-4* strain.

Taken together, our results show that the catalytic core of the PI3K complex, namely subunits Vps15 and Vps34, is essential for αSyn-GFP localization at the PM, as reintroducing either gene rescues the mislocalization and cytoplasmic accumulations. By contrast, deletions of other complex subunits (*e.g.*, Atg6, Atg14, Atg38, Vps38) or distinct PIP kinases, which do not alter cellular PI3P levels, have no influence on αSyn-GFP localization.

### αSyn-GFP enters the secretory pathway *via* PI3P and colocalizes with Sec4

We have shown that PI3P synthesis is necessary for PM localization of WT and A53T αSyn-GFP in yeast. Recent research indicates that a functional secretory machinery is required for the intracellular transport of human αSyn to the PM (21, 24) and that yeast deletion strains of genes required for vesicular trafficking pathways are more sensitive to αSyn expression (26). Consequently, we hypothesized that αSyn might enter the secretory pathway *via* its interaction with PI3P.

**Figure 5:**
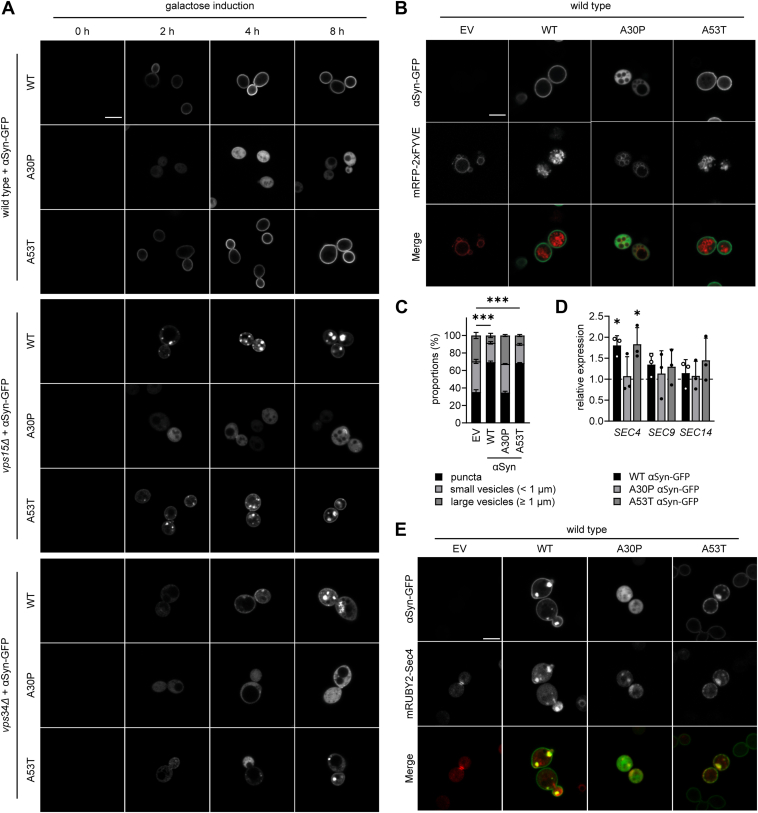
**PM-binding αSyn-GFP influences the yeast secretory pathway and colocalizes with Sec4.** All experiments were conducted with cells expressing galactose-inducible αSyn-GFP constructs. *A*, localization of WT, A30P, and A53T αSyn-GFP over time after induction of gene expression in wild type, *vps15Δ,* and *vps34Δ* strains. In the wild type, WT and A53T αSyn-GFP localize to budding sites after 2 h of expression. In the *vps15Δ* and *vps34Δ* strains, these variants are localized in the cytoplasm early after induction and form large accumulations. The scale bar represents 5 μm. *B*, localization of PI3P in wild type strains expressing WT, A30P, and A53T αSyn-GFP. The scale bar represents 5 μm. *C*, quantification of puncta, small vesicles (<1 μm), and larger vesicles (≥1 μm) from (*B*). Expression of WT and A53T αSyn-GFP in wild type strains increases relative amount of small PI3P-rich structures from 60 to 90%. Results are shown as mean ± standard deviation. N = 3, with at least 100 cells per N. *D*, quantitative real-time PCR analysis of *SEC4*, *SEC9,* and *SEC14* mRNA levels in the wild type strain expressing αSyn-GFP. Expression of WT and A53T αSyn-GFP almost doubles SEC4 expression in wild type strains. C_T_ values are normalized to the EV control. *C*–*D*, ∗*p* < 0.05 and ∗∗∗*p* < 0.001. *E*, colocalization of overexpressed mRUBY2-Sec4 from a one-copy plasmid with different αSyn-GFP isoforms. mRUBY2-Sec4 colocalizes with WT and A53T αSyn-GFP in large accumulations close to the budding sites. The scale bar represents 5 μm. αSyn, alpha-synuclein; PI3P, phosphatidylinositol 3-phosphate; EV, empty vector; PM, plasma membrane.

Initially, we monitored the distribution of the different αSyn-GFP isoforms over time, using a galactose-inducible expression system. In line with previous findings using N terminally tagged GFP-αSyn constructs in wild type strains (21), we confirmed that our C terminally tagged WT and A53T αSyn-GFP isoforms localize to the PM at the budding sites 2 h after induction of expression and distribute throughout the whole PM after 4 h of induction (Fig. 5*A*). This confirms that αSyn-GFP is likely to be transported *via* the secretory machinery in wild type strains. A30P αSyn-GFP, on the other hand, localizes to the cytoplasm already 2 h after galactose induction. Similarly, in *vps15Δ* and *vps34Δ*, αSyn-GFP is primarily localized in the cytoplasm already after 2 h of expression. Notably, in the *vps34Δ* strain, cytoplasmic accumulations of αSyn-GFP do not appear until 8 h after induction, whereas in the *vps15Δ* strain, accumulation begins as early as 2 h after galactose addition. This could be explained by the comparatively slower metabolism of *vps34Δ*, which may delay the response to changes in carbon source (65).

As the transport of PI3P is critical for a functional secretory pathway (31, 66) and the absence of the PI3K Vps34 causes mislocalization of αSyn-GFP in yeast, we investigated whether the expression of αSyn-GFP isoforms might cause changes in the amount and distribution of PI3P-containing vesicles. To this end, we analyzed mRFP-2xFYVE–positive structures upon expression of the WT, A30P, and A53T αSyn-GFP constructs. Indeed, we found that WT and A53T αSyn-GFP expression causes an accumulation of small mRFP-positive puncta and vesicles that make up approximately 90% of PI3P-containing structures. In contrast, the expression of A30P αSyn-GFP does not change the distribution of PI3P-containing vesicles and structures when compared with the EV control (Fig. 5, *B* and *C*). The substantial increase in PI3P-containing puncta upon expression of WT and A53T αSyn-GFP may indicate a mobilization of endosomal vesicles in order to accelerate secretory transport to the PM (31).

To understand this, we analyzed mRNA expression levels of the endogenous secretory genes *SEC4*, *SEC9*, and *SEC14*. We chose to analyze the Rab GTPase Sec4 and the PM-localized t-SNARE protein Sec9, as they are directly involved in targeting and fusion of secretory vesicles at the developing cell bud, respectively (67, 68, 69). Sec14, on the other hand, is a cytosolic PL transport protein mainly found in intra-Golgi trafficking pathways (70) and is not directly involved in secretory vesicle fusion at the PM. Interestingly, expression of WT and A53T αSyn-GFP, but not A30P αSyn-GFP, from a one-copy *CEN/ARS* plasmid, increases *SEC4* expression 2-fold (Fig. 5*D*), whereas *SEC9* and *SEC14* transcript levels are not significantly changed. Thus, we conclude that the expression of membrane-binding αSyn-GFP isoforms (WT and A53T) promotes the accumulation of small PI3P-enriched vesicles and specifically upregulates the secretory pathway component *SEC4*, which facilitates vesicle targeting to the PM. In contrast, A30P αSyn-GFP, lacking lipid-binding properties, does not induce these effects.

We next investigated the intracellular localization of Sec4, Sec9, and Sec14 in cells expressing WT, A30P, or A53T αSyn-GFP. To this end, we coexpressed N terminally tagged mRUBY2 fusion constructs from a one-copy CEN/ARS plasmid with each αSyn-GFP isoform and analyzed the localization of each protein *via* microscopy. Interestingly, only mRUBY2-Sec4 colocalizes with WT and A53T αSyn-GFP, inducing the formation of large αSyn- and Sec4-containing puncta near emerging PM-budding sites (Fig. 5*E*). This effect is not observed for mRUBY2-Sec9 and mRUBY2-Sec14 (Fig. S7). This suggests a possible direct interplay between mRUBY2-Sec4 and membrane-binding αSyn-GFP in the terminal steps of vesicle secretion for cell budding. Moreover, the formation of large puncta suggests that co-overexpression of these proteins may hinder efficient vesicle-PM fusion.

## Discussion

The pathological aggregation of αSyn and the subsequent formation of Lewy bodies in progressive PD is not solely an amyloidogenic process, but rather an accumulation of protein fibrils and membrane compartments, such as vesicles and small organelles (19). *In vitro* aggregation studies have shown that the presence of membrane lipids can enhance the primary nucleation rate of αSyn (71). Therefore, elucidating interactions between PLs and αSyn is crucial for understanding disease formation and progression. In the past, using *S. cerevisiae* as a model to investigate basic cellular mechanisms that are affected by expressing human αSyn has proven to be a versatile tool [reviewed in (72, 73)]. Here, our initial goal was to describe dose-dependent effects of human WT αSyn and the PD-related isoforms A30P and A53T αSyn on cell growth as well as on the intracellular quality control mechanisms autophagy and proteasomal degradation.

We found that high expression levels of WT and A53T αSyn-GFP induce the aggregation of endogenous proteins, without any substantial influence on autophagic activity. It is not surprising, however, that autophagy levels remain unchanged, and that the growth of the *atg1Δ* strain is unaffected in our experiments, given that our analyses were performed in exponentially growing cells. Past studies have shown that αSyn expression induces autophagy primarily in aged, stationary-phase yeast cells, whereas in young cells, only Atg8 expression levels increase, without significant changes in autophagic flux (27, 30). This supports our observation that ATG effects are minimal under our experimental conditions. Notably, the *atg1Δ* strain has been reported to exhibit a significantly shortened lifespan even in the absence of αSyn, underscoring the role of Atg1, especially in long-term cellular stress response (74). On the other hand, our data shows that the 26S proteasome is significantly inhibited by the expression of WT αSyn-GFP alone. This seems to contradict a study from Outeiro and Lindquist in 2003, which showed that expression of all three αSyn isoforms inhibits the *in vivo* degradation of ubiquitinated GFP, a reporter protein for the ubiquitin-proteasome system (20). It is important to note, however, that the 26S proteasome assay specifically measures chymotrypsin-like activity, whereas Outeiro and Lindquist (2003) assessed global ubiquitin-dependent protein turnover in living cells. Thus, while only WT αSyn appears to impair 26S catalytic activity in our study and others (37), all three αSyn variants are likely to inhibit upstream processes of the general proteasome machinery. Indeed, a recent study showed that WT αSyn specifically inhibits 26S activity by interacting with the proteasomal assembly chaperone Rpn14 (37). For future studies, it will be interesting to see whether the A30P and A53T isoforms can interact with Rpn14 and, as a result, might not affect the 26S core activity.

Similar to the results observed in the *atg1Δ* strain, expression of αSyn-GFP from a one-copy construct does not affect most *ATG* deletion strains. Our growth screenings and subsequent microscopic analyses demonstrate that deletion strains deficient in specific PIP metabolism pathways are especially sensitive to αSyn-GFP expression. Further investigations have shown that PM localization of human αSyn-GFP *via* the secretory pathway depends on two key factors: an intact membrane-binding N terminus of αSyn and functional PI3P biosynthesis, facilitated by the yeast’s only PI3K, Vps34. Specifically preventing PI3K complex I assembly at the pre-autophagosomal structure by deleting *ATG14* demonstrates that autophagosomal PI3P is not required for PM localization. However, a loss of PI3K catalytic activity itself results in the cytoplasmic accumulation of membrane-binding WT and A53T αSyn-GFP and the presence of FM4-64–positive lipids in these accumulations suggests defects in membrane-driven αSyn-GFP transport. Although our thioflavin T stainings did not indicate any formation of αSyn-GFP amyloid aggregates in the PI3K deletion strains, investigating αSyn accumulations caused by membrane transport defects remains highly relevant. Studies with wild type yeast strains expressing high levels of αSyn-GFP have shown nonamyloid accumulations similar to those in our PI3K mutants. These accumulations contained large amounts of small vesicles and colocalized with Rab GTPases Sec4 and Ypt1 (25). In PD patients, αSyn pathology is also not exclusively attributed to amyloid aggregation. Indeed, in PD neurons, pale bodies and specific Lewy body–related structures were found to contain membranes, whole organelles, and, most importantly, nonfibrillar αSyn (19, 75, 76). These studies suggest that αSyn pathology classifies as both proteinopathy and a lipidopathy.

αSyn is well-known to target the yeast cell bud *via* the secretory machinery. Dixon *et al.* (2005) have shown that temperature-dependent inactivation of essential secretory genes *SEC4*, *SEC9*, *SEC12*, *SEC14*, and *SEC23* causes a mislocalization of αSyn in the cytoplasm (21). Also, the overexpression of the Rab GTPase Ypt1, involved in endoplasmic reticulum–Golgi vesicle trafficking, rescues αSyn toxicity in yeast (77). However, a specific entry point for αSyn into this complex machinery has yet to be defined. PI3P is synthesized at the early endosome by the yeast PI3K complex II and is subsequently targeted to either multivesicular bodies (58) or the Golgi apparatus *via* the retromer complex and the sorting nexins Atg24 (Snx4) and Atg20 (Snx42) (31). Most likely, αSyn is transported to the Golgi *via* endosomal, PI3P-rich vesicles. This is supported by a recent analysis showing that αSyn competes with Atg24 for binding to PI3P-containing vesicles (78). Interestingly, the PX domains of the Atg24 dimer specifically target liposomes containing PI3P or PI(3,5)P2 species, whereas Atg20 binds to PI and PI4P species as well (46). This aligns with our ATG screening, showing that only the loss of Atg20, but not Atg24, affects growth and PM localization of αSyn-GFP. In the *atg20Δ* strain, the absence of Atg20 requires compensation by Atg24 for endosome-Golgi transport. Since Atg24 is blocked by αSyn from binding to PI3P-containing vesicles, retromer activity would be impaired. Conversely, the deletion of *ATG24* leaves Atg20 as the critical sorting nexin for retromer transport. The ability of Atg20 to bind to different PIP species might be sufficient to stabilize endosome-Golgi transport, subsequently promoting PM localization of αSyn. In addition, deletion of Ypt6, a Rab GTPase necessary for endosome-Golgi trafficking alongside the retromer complex causes mislocalization and cytoplasmic accumulation of αSyn, similar to our observations in the Vps34 deletion mutant (24). Taken together, both of these findings imply that the transport of αSyn-GFP to the PM is dependent on a functional endosome-Golgi transport mechanism.

Since PI3P-rich vesicles are not directly trafficked to the PM, the transport of αSyn to the PM most likely occurs *via* the recycling component of the endosomal trafficking pathway (58). Canonically, at the Golgi, PI4P species–containing vesicles are transported to the PM *via* the interaction of Rab GTPases and SNARE proteins with the PM-localized exocyst complex (79, 80, 81). However, in our study, the absence of Golgi PI4P biosynthesis or the absence of other PI4-related kinases does not show severe effects on protein localization. We found that deletion or activity reduction of PI4Ks Lsb6 or Stt4, respectively, causes αSyn to accumulate after reaching the PM *via* the secretory pathway. In contrast, the loss of PI(3,5)P2-synthesizing Fab1 (PIKfyve in mammals) results in αSyn mislocalization similar to the one observed in the *vps15Δ* strain. Interestingly, lipid overlay assays with αSyn have shown that αSyn binds to PI3P and PI(3,5)P2 with a much higher affinity than to PI4P and PI(4,5)P2 (34). A recent study demonstrated that PIKfyve, and therefore PI(3,5)P2 biosynthesis, plays an important role in regulating targeting of secretory vesicles from the endosome to the PM *via* the SNX17–Retriever–CCC–WASH complex (66, 82, 83). Moreover, human PIKfyve has been shown to associate with RAB11A, a Rab GTPase involved in the recycling of endosomal vesicles to the PM (66). In yeast cells, high amounts of αSyn accumulate in transport vesicles and colocalize with the RAB11A ortholog Ypt31 (84). A putative PIKfyve-dependent PM translocation of αSyn is supported by the fact that overexpression of inositol polyphosphate 5-phosphatase in human ovarian adenocarcinoma A2780 cells led to a decrease in PM-localized αSyn (33). Similarly, a direct endosomal recycling pathway could, at least partially, transport αSyn from endosomes to the PM. Thus, further lipidomic analyses of PIP levels in purified PM fractions of strains expressing αSyn are necessary to elucidate differences in PM composition (85).

We also demonstrated that lipid-binding isoforms of αSyn induce expression of endogenous Sec4, an important factor for targeting endosomal vesicles to the exocyst at newly emerging cell buds. This finding was surprising, considering that αSyn is not an endogenous protein in *S. cerevisiae*. The mRNA enrichment caused by αSyn has been confirmed on the protein level by a recent proteomic analysis, which demonstrated that WT αSyn increases cellular Sec4 levels significantly, and that both proteins localize in close proximity to each other (29). Unsurprisingly, we found that αSyn (WT and A53T) strongly colocalizes with Sec4 at the emerging bud, and overexpressing both proteins causes a colocalized accumulation of both proteins. Strikingly, Sec4 is a functional ortholog of human Rab GTPases, such as RAB3A (51.7% identity), which is known to be an important regulator for the priming and secretion of synaptic vesicles at the presynapse (86). Sec4 associates with the v-SNARE protein Snc2 on secretory vesicles. Snc2 is the yeast ortholog of mammalian VAMP2, a synaptic interactor of RAB3A, and necessary for targeting synaptic vesicles to the SNARE complex (87). Similar to Sec4, Snc2 is upregulated by αSyn expression in *S. cerevisiae* (29). At the PM, Snc2 engages the t-SNARE Sec9 together with Sso1 or Sso2, orthologous to SNAP-25 and syntaxins, respectively, thereby mirroring the docking mechanism at the neuronal SNARE complex (67, 68, 88).

*In vitro* studies have shown an interaction between αSyn and human RAB3A *via* its C-terminal domain, which strengthens on the surface of lipid membranes (89). This is especially interesting considering that only membrane-binding αSyn colocalizes with Sec4 in the yeast model. Sequence alignments also point toward a potential protein–protein interaction in yeast, as the αSyn-binding domain of RAB3A shares approximately 82% sequence identity with the corresponding region in the yeast Sec4 protein (89). However, our data indicate that colocalization of αSyn and Sec4 results from both proteins binding to the same vesicles. This is supported by studies demonstrating that C terminally truncated αSyn still localizes to the yeast PM (90), and that the N terminally truncated variants in our study are not membrane-associated. This is consistent with observations in mammalian systems, where C terminally truncated αSyn still targets the PM and interacts with the plasma membrane calcium ATPase (91). Similarly, in mouse striata, truncated αSyn (1–120 aa) remains associated with the t-SNARE SNAP-25 at presynaptic terminals (92). It also suggests that binding to specific membrane lipids, such as PI3P, plays an additional role in PM localization other than direct interaction with Rab GTPases.

In our yeast model, binding of αSyn and Sec4 to the same vesicles might also explain why Sec4 accumulates at the cell bud, whereas Sec9 does not. Sec9 functions as a PM-localized t-SNARE and therefore does not directly associate with the trafficked vesicles bound by αSyn. Consequently, αSyn expression does not directly affect the membrane fusion efficiency mediated by Sec9. Sec4 targeting the same αSyn-bound vesicles, however, may compete in binding or overload the surface of those vesicles. This could subsequently impair interaction with the exocyst complex and ultimately hinder vesicle docking and fusion processes at the PM.

**Figure 6:**
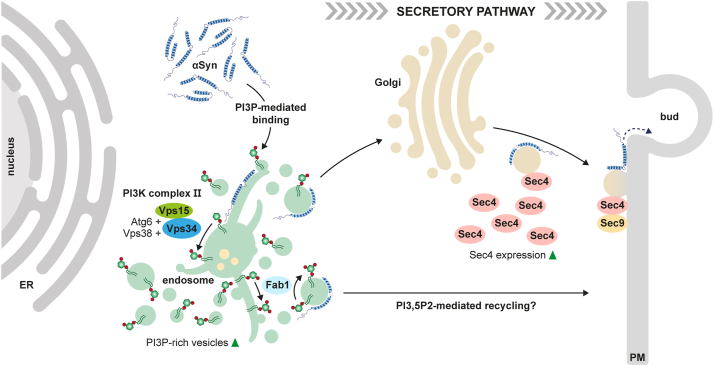
**Proposed model for αSyn trafficking to the yeast PM.** Heterologous expression of αSyn promotes the increased formation of small PI3P-enriched vesicular structures, likely corresponding to endosomal compartments. We hypothesize that αSyn binds to these PI3P-positive vesicles, which are generated at endosomes by the PI3K complex II. From here, αSyn potentially follows two nonmutually exclusive trafficking routes: a direct recycling pathway to the PM that involves PI(3,5)P2 signaling, though the specific mechanism remains to be elucidated; or retrograde transport to the Golgi apparatus. At the trans-Golgi network, αSyn could subsequently be transported to the PM through interaction with the exocyst complex. This fusion is most likely mediated by the Rab GTPase Sec4 (RAB3A ortholog), which is transcriptionally upregulated by αSyn expression. αSyn, alpha-synuclein; PM, plasma membrane; PI3K, PI3 kinase; PI3P, phosphatidylinositol 3-phosphate.

In the past, many studies have demonstrated a high variability of cellular αSyn localizations and proposed putative physiological roles, such as transcriptomic regulation and synaptic vesicle trafficking, as well as dose-dependent pathological effects such as aggregation and cytotoxicity [reviewed in (93)]. Overall, our study provides an improved mechanistic understanding of human αSyn-localization to the PM of *S. cerevisiae via* the secretory pathway (summarized in Fig. 6). Unlike mammalian systems that possess multiple isoforms, *S. cerevisiae* has only one PI3K, Vps34. Therefore, we could eliminate PI3K activity entirely and monitor αSyn trafficking to the PM. Our findings provide the first evidence that PI3P-specific binding of αSyn to endosomal membranes might act as an entry point into the highly conserved yeast secretory pathway, and by extension, into equivalent trafficking routes in other eukaryotic systems (72, 94). In mammalian cells, αSyn interaction with PI3P could also be a possible determinant for its association with vesicles that transport it to the axon terminal. This idea is supported by observations that N terminally truncated αSyn variants, as well as the A30P mutant, are delivered to the axon terminal at significantly reduced rates (95). In addition, the presence of PI3P on synaptic endosomes may help recruit αSyn to recycled synaptic vesicles. This is consistent with studies showing that synaptic vesicle recycling *via* the endosomal pathway is PI3P-dependent and that the mammalian PI3K-activating Rab GTPase RAB5 associates with αSyn at the synapse (96, 97, 98).

In *S. cerevisiae*, the final stages of secretion toward the emerging bud closely resemble synaptic SNARE complex assembly in neurons. The interaction of human αSyn with yeast orthologs of synaptic PM fusion factors underscores the model’s relevance for PD research (87). This is why, despite the phylogenetic distance between yeast and mammals, meaningful parallels can be drawn, particularly in the investigation of αSyn-transporting vesicles. The yeast model, therefore, not only enables the identification of specific protein and lipid factors involved in αSyn localization but also provides valuable insights into molecular interfaces, such as PI3P-mediated entry into PM-targeting transport mechanisms, that may represent potential targets for relevant pathological changes in the context of PD. As no current model system involving the full spectrum of PD-related processes exists, it is crucial to focus on isolated mechanisms with the intention of translating the gained knowledge to other systems in the future.

## Experimental procedures

### Yeast strains and cultivation

All *S. cerevisiae* strains used in this study were derived from the BY4741 “wild type” (99). The full genotypes of all mutants are listed in Table S1. If not stated otherwise, all cells were grown to log phase (OD_600__≈_ 0.4–0.8) at 30 °C, shaking at 180 rpm in minimal synthetic defined (SD) medium containing 0.67% (w/v) yeast nitrogen base without amino acids and 2% (w/v) glucose, supplemented with 1 mM L-histidine, 1 mM L-leucine, 0.5 mM L-methionine, and 0.2 mM L-uracil. For yeast transformation (100), strains were grown either in SD medium or yeast extract peptone dextrose medium, containing 2% (w/v) bacto peptone, 1% (w/v) yeast extract, and 2% (w/v) glucose. For nitrogen starvation, we used SD-N medium containing 2% glucose and 0.17% (w/v) yeast nitrogen base without amino acids and ammonium sulfate (101). To induce gene expression under the control of a *GAL1* promoter, all strains were grown on SD medium containing 2% raffinose as carbon source. Gene expression was induced by adding 2% galactose to the culture. Cultivation for each experiment was performed at 30 °C.

### Cloning procedure for plasmid generation

The plasmids used in this work are listed in Table S2. We used *CEN/ARS* and 2 μ containing plasmid backbones for one-copy and high-copy plasmid counts, respectively. Plasmids were constructed using standard cloning procedures and commercially available restriction enzymes (New England Biolabs GmbH). The WT, A30P, and A53T αSyn-GFP constructs on 2 μ backbones have been described previously (20). The mRFP-2xFYVE reporter and the DEG1-SEC62 construct were kindly provided by Prof. Dr Thumm and Prof. Dr Rubenstein, respectively.

### Growth assessments

Growth curves were obtained by cultivating the yeast strains in SD selective medium until stationary phase and subsequent dilution to OD_600_ = 0.1 in fresh medium. The strains were grown for up to 16 h in a “Varioskan Lux” shaker (Thermo Fisher Scientific) at 30 °C with 180 rpm. For growth comparisons, we evaluated the highest growth rate in the log phase of each strain.

### Fluorescent microscopy

To image fluorescently tagged proteins in our yeast strains, cells were harvested by centrifugation and washed with potassium buffered saline (PBS). Cell suspensions were spotted on concanavalin A–coated microscope slides. The rims around the coverslips were sealed with nail polish. For fluorescent imaging, we used the confocal laser scanning microscope “Zeiss LSM710” (Zeiss) with a 1000× magnification. For quantifications, at least 100 cells per biological replicate were included. For FM4-64 staining, 0.8 mM FM4-64 (T13320, Thermo Fisher Scientific) was added to exponentially growing yeast cells and incubated for 30 min at 30 °C. Unbound dye was replaced by fresh medium and cells were incubated for additional 60 min at 30 °C before microscopy. In order to stain cells for potential amyloid aggregates, thioflavin T (88630, Honeywell Fluka) was used. For this, cells were fixed in 4% paraformaldehyde (in PBS) and treated with 30 μM thioflavin T in PBS (+0.1% Triton X-100) for 30 min at room temperature. Microscopy of Stt4-inhibited cells was performed by adding 3 μM Wortmannin (W1628, Sigma-Aldrich) in dimethyl sulfoxide to the growing cells 5 h before imaging.

### TCA-precipitation and immunoblotting

Total cell lysates were achieved by standard trichloroacetic acid (TCA) precipitation (102). For this, 20 ODs of yeast culture were harvested by centrifugation and mechanically disrupted with glass beads in 10% TCA. Crude lysates were washed twice with ice cold acetone, dried, and resuspended in resuspension buffer at 37 °C. Samples were mixed with protein sample buffer, denatured at 95 °C, and separated *via* SDS-PAGE in 12% Bis-Tris gels. Protein transfer on nitrocellulose membranes for immunoblotting was performed *via* the Trans-Blot Turbo transfer system (Bio-Rad, Hercules), according to manufacturer’s instructions. For immunological protein detection, the following primary antibodies were used: monoclonal mouse anti-GFP (SAB5300167, Sigma-Aldrich) and monoclonal anti-Pgk1 (459250, Thermo Fisher Scientific). As secondary antibody horseradish peroxidase-conjugated monoclonal donkey anti-mouse antibody (715-035-151, BIOZOL) was used. Chemiluminescent signals were detected by an Amersham Imager 600 (GE Healthcare). Videodensitometric quantification was performed with the Image Studio Lite software v5.2 (LI-COR, https://www.licorbio.com/image-studio-lite).

### Quantitative real-time PCR

For total RNA isolation 20 ODs of mid-log phase cells were used. RNA extraction was performed using TRIzol reagent (15596026, Thermo Fisher Scientific) following the manufacturer's protocol. Briefly, yeast cells were harvested, and the cell pellet was mechanically disrupted with glass beads in TRIzol reagent. After phase separation by addition of CHCl_3_, the aqueous phase containing RNA was collected and precipitated with isopropanol. The RNA pellet was washed with 75% ethanol (v/v) and resuspended in RNase-free water. The RNA concentration was determined using a spectrophotometer at 260 nm, and the purity was assessed by the A260/A280 ratio.

DNase-treated RNA (1 U/μg RNA) was reverse transcribed into complementary DNA according to the “Omniscript RT Kit” (Qiagen) protocol. The primers used for quantitative real-time PCR are listed in Table S3. Expression levels of selected genes were normalized to *ACT1*. For all genes, PCR efficiency was determined as described in Pfaffl (2001) (103).

### CHX chase

CHX chase was performed according to Buchanan *et al.* (2016) (38) with minor alterations. For each time point 2.5 ODs of mid-log phase cell suspension were harvested and resuspended in fresh SD medium. Each culture was pre-warmed in a water bath at 30 °C. At t_0_, 250 μg/ml CHX (8682.1, Carl Roth) was added to the samples. At each time point, cells were harvested, mixed with 20× Stop mix (200 mM NaN_3_, 5 mg/ml bovine serum albumin, and put on ice. After the assay, the samples were washed with 10% TCA and prepared for SDS-PAGE and immunoblotting as already described.

### Autophagic flux assay

Autophagic flux in nitrogen-starved cells was measured according to a previously described *Pho8Δ60* assay (36). Briefly, cells were grown to log phase and shifted to SD-N medium. After 16 h incubation at 30 °C the cells were washed with 0.85% NaCl (+1 mM PMSF) and mechanically disrupted with glass beads in lysis buffer (20 mM Pipes, 0.5% Triton X-100, 50 mM KCl, 100 mM KAc, 10 mM MgSO_4_, 10 μM ZnSO_4_, 1 mM PMSF). Phosphatase activity was measured in reaction buffer (250 mM Tris/HCl, pH 8.5, 0.4% Triton X-100, 10 mM MgSO_4_, 10 μM ZnSO_4_) by the turnover of p-nitrophenyl phosphate (N1891, Sigma-Aldrich) to p-nitrophenol (absorbance at 400 nm) at 37 °C. The reaction was stopped using 1 M glycine/KOH, pH 11. The specific activity was calculated as A_400_/min/mg protein.

### 26S proteasome assay

Chymotrypsin-like proteasome activity was determined as previously published (37). The strains were grown to log phase in SD selective medium. Cells were harvested by centrifugation and mechanically disrupted by glass beads in lysis buffer (50 mM Tris/HCl, pH 7.5, 100 mM NaCl, 2 mM DTT, 2 mM ATP, 5 mM MgCl_2_). Protein concentration was determined by the bicinchoninic acid assay and 60 μg of yeast protein were used for each analysis. The activity of the 20S proteasome was measured using 100 μM of the fluorogenic substrate Succinyl-Leu-Leu-Val-Tyr-7-amino-4-methylcoumarin (ENZOBMLP8020005, Enzo Life Sciences) in reaction buffer (20 mM Tris/HCl, pH 7.5, 50 mM NaCl, 2 mM DTT). AMC release was measured for 1 h at 30 °C (excitation: 350 nm; emission: 440 nm). The maximum turnover rate was used for comparative analysis.

### Aggregated protein assay

The yeast aggregated protein assay was performed according to Weids and Grant (2014) (104). Briefly, all strains were grown in selective media until exponential growth and sedimented *via* centrifugation. Cells were washed with aggregate lysis buffer (50 mM potassium phosphate, 1 mM EDTA, 5% [w/v] glycerol, 1 mM PMSF, and 1× c0mplete protease inhibitor cocktail [Roche]). Cell lysis was performed with 12.5 U/ml Lyticase for 30 min at 30 °C, and subsequent sonification. Aggregated fractions were obtained by centrifugation and multiple washing steps with aggregate lysis buffer with 2% IGEPAL CA-630. The aggregated fractions were visualized *via* SDS-PAGE and the SilverXpress silver stain kit with instructions of the manufacturer (Invitrogen).

### Statistical analysis

Statistical analysis was performed either by a two-tailed Students *t* test or a one-way ANOVA with *post hoc* analysis. ∗*p* < 0.05, ∗∗*p* < 0.01, and ∗∗∗*p* < 0.001. Error bars in all figures represent standard deviations.

## Data availability

All data are contained within this article.

## Supporting information

This article contains supporting information (20, 38, 105, 106). Supporting Figures S1-S7 and Tables S1-S3 are available online.

## Conflict of interest

The authors declare that they have no conflicts of interest with the contents of this article.
