# Supplementary material for: Phosphatidylinositol 3-phosphate metabolism impacts cellular α-synuclein localization in Saccharomyces cerevisiae
Source: J Biol Chem. 2025 Sep 1;301(10):110666. doi: 10.1016/j.jbc.2025.110666 (PMC12514572; doi:10.1016/j.jbc.2025.110666)
Supplement: Supporting Figures S1-S7 [file mmc1.pdf]

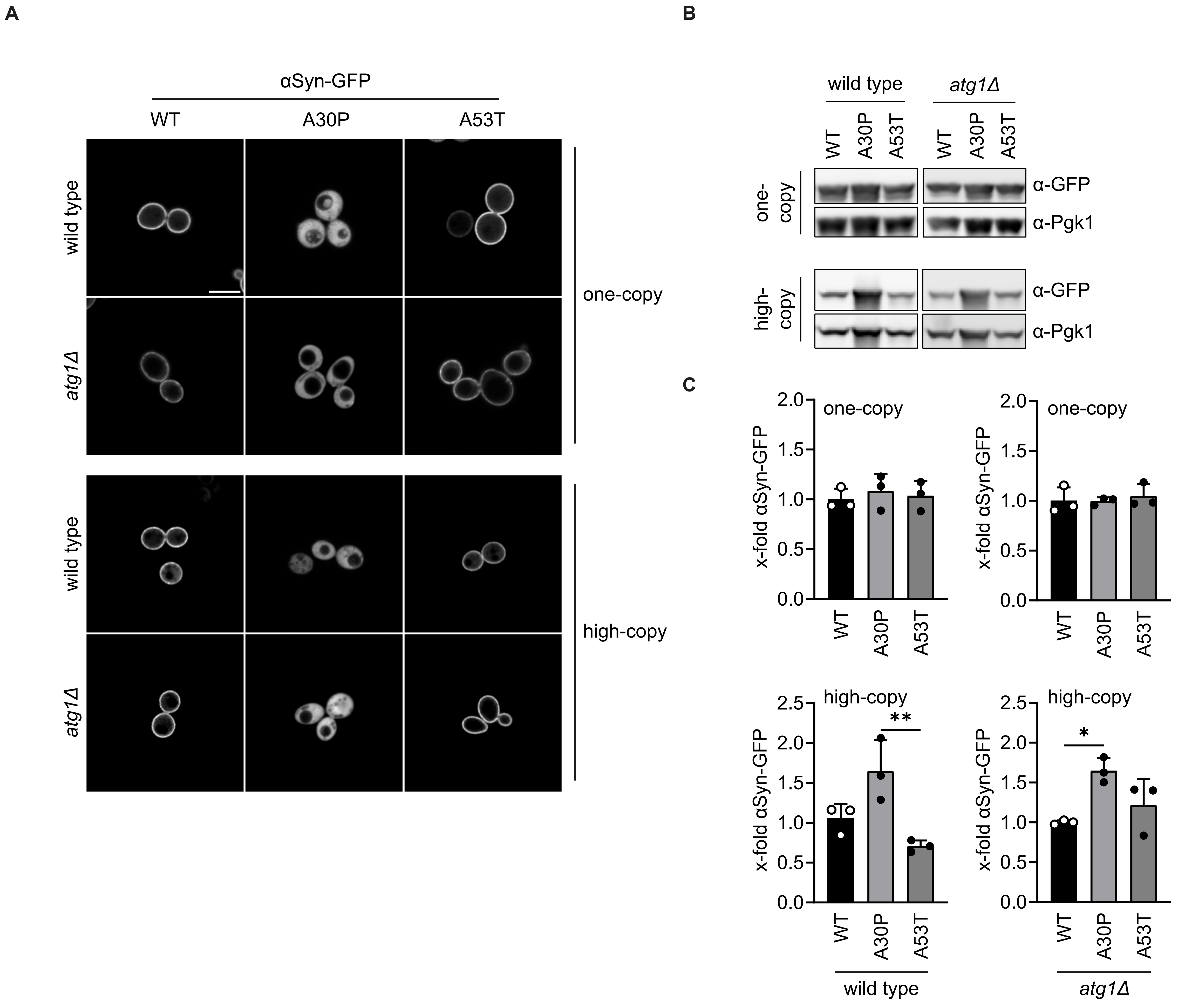

Figure S1: High-copy WT, A30P, and A53T αSyn-GFP constructs show different expression levels. A: Fluorescence microscopy of wild-type and *atg1Δ* strains expressing WT, A30P, and A53T αSyn-GFP from one-copy or high-copy plasmids. WT and A53T αSyn-GFP localizes to the plasma membrane, whereas the A30P variant is cytoplasmic. Scale bar: 5 µm. **B:** Western Blot analysis and **C:** quantification of expression levels of WT, A30P, and A53T αSyn-GFP expressed from one-copy (top) or high-copy (bottom) plasmids in wild-type and *atg1Δ* strains. The high-copy constructs show different αSyn-GFP expression levels, while expression from the one-copy plasmids is stable. Values represent mean + standard deviation. N = 3; *p < 0.05; **p < 0.01.

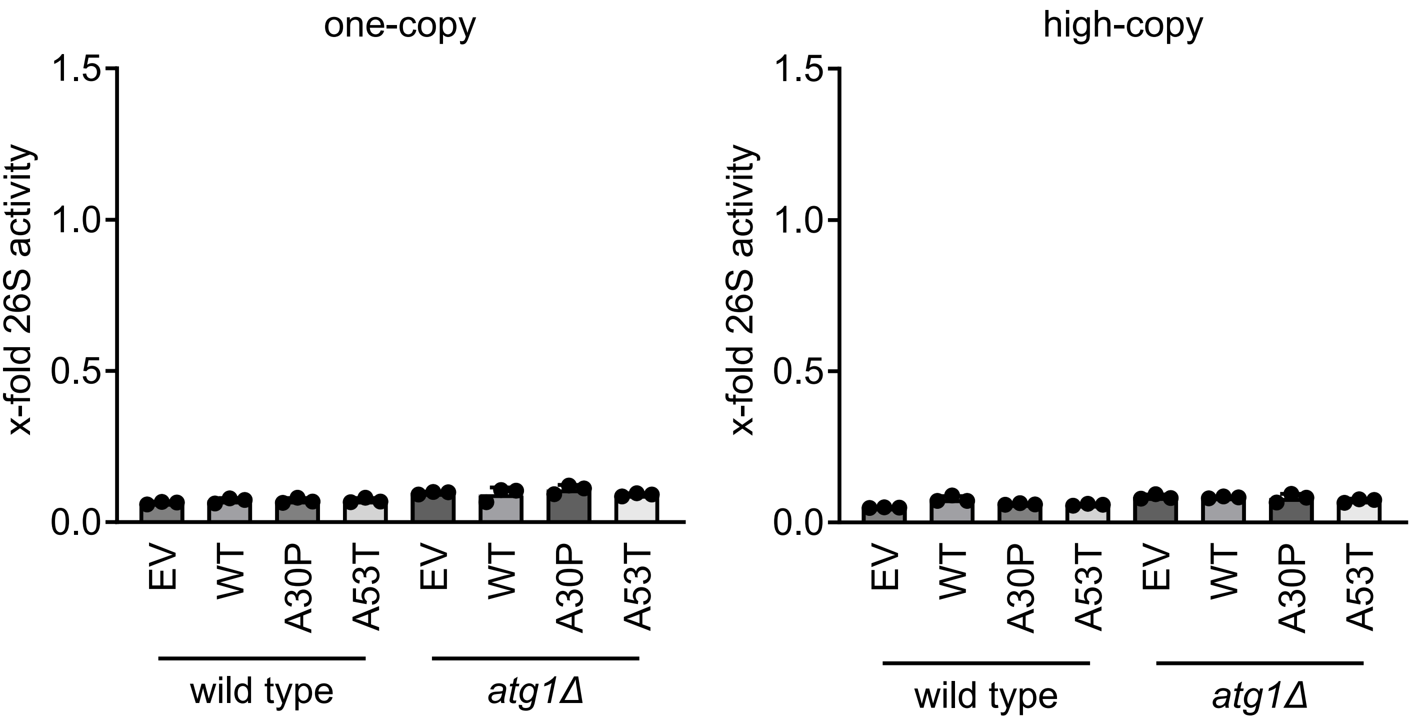

Figure S2: MG-132 inhibits proteasomal activity in wild-type and *atg1Δ* strains. Relative proteasomal activity of wild-type and *atg1Δ* strains expressing WT, A30P, and A53T αSyn-GFP one-copy (left) and high-copy (right) constructs. The low activity values after MG-132 treatment confirm that the experiment in Figure 1C is specific for 26S activity. Values are normalized to the wild-type EV control in Figure 1C and represent mean + standard deviation. N = 3.

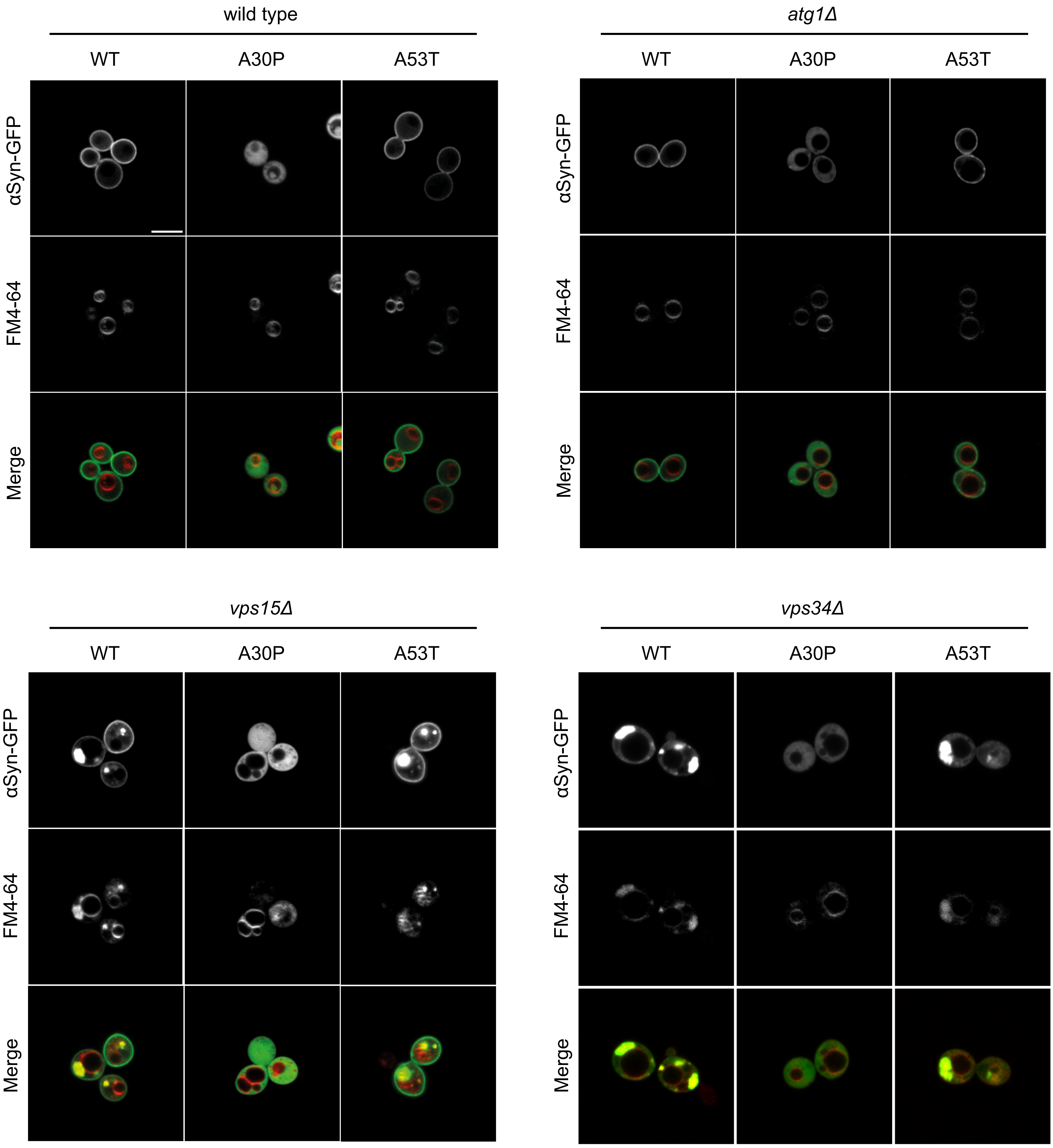

Figure S3: Accumulations of αSyn-GFP contain membrane species. FM4-64 straining of wild-type, *atg1Δ*, *vps15Δ*, and *vps34Δ* strains expressing αSyn-GFP reveal that accumulations of WT and A53T αSyn-GFP contain membrane lipids. Scale bar: 5 µm.

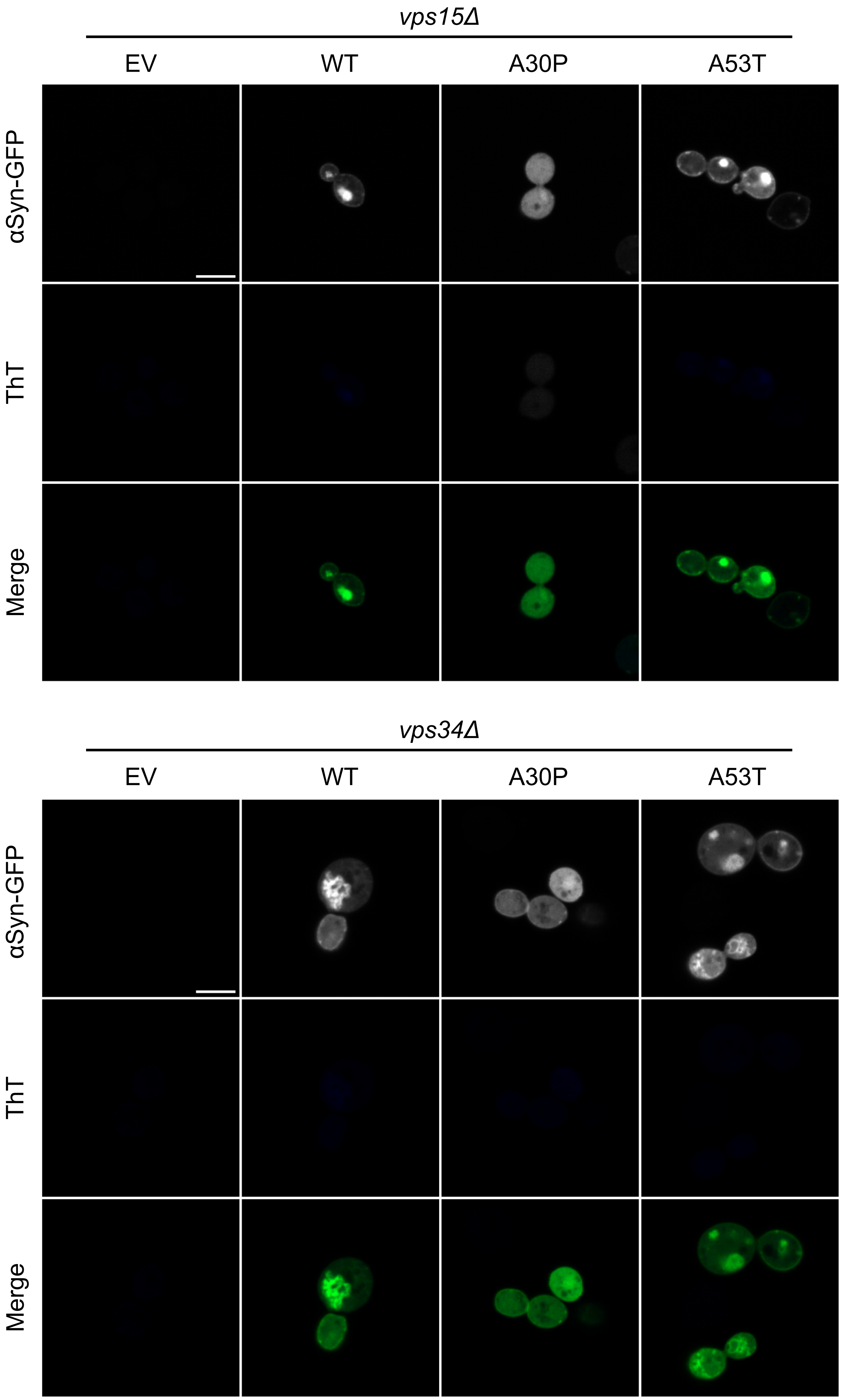

Figure S4: Cytoplasmic αSyn-GFP accumulations in *vps15Δ* and *vps34Δ* are non-amyloid. Thioflavin T (ThT) staining of *vps15Δ* and *vps34Δ* expressing WT, A30P, and A53T αSyn-GFP. All observed cells are negative for ThT, and therefore, contain no amyloid αSyn-GFP aggregates. Scale bar: 5 µm.

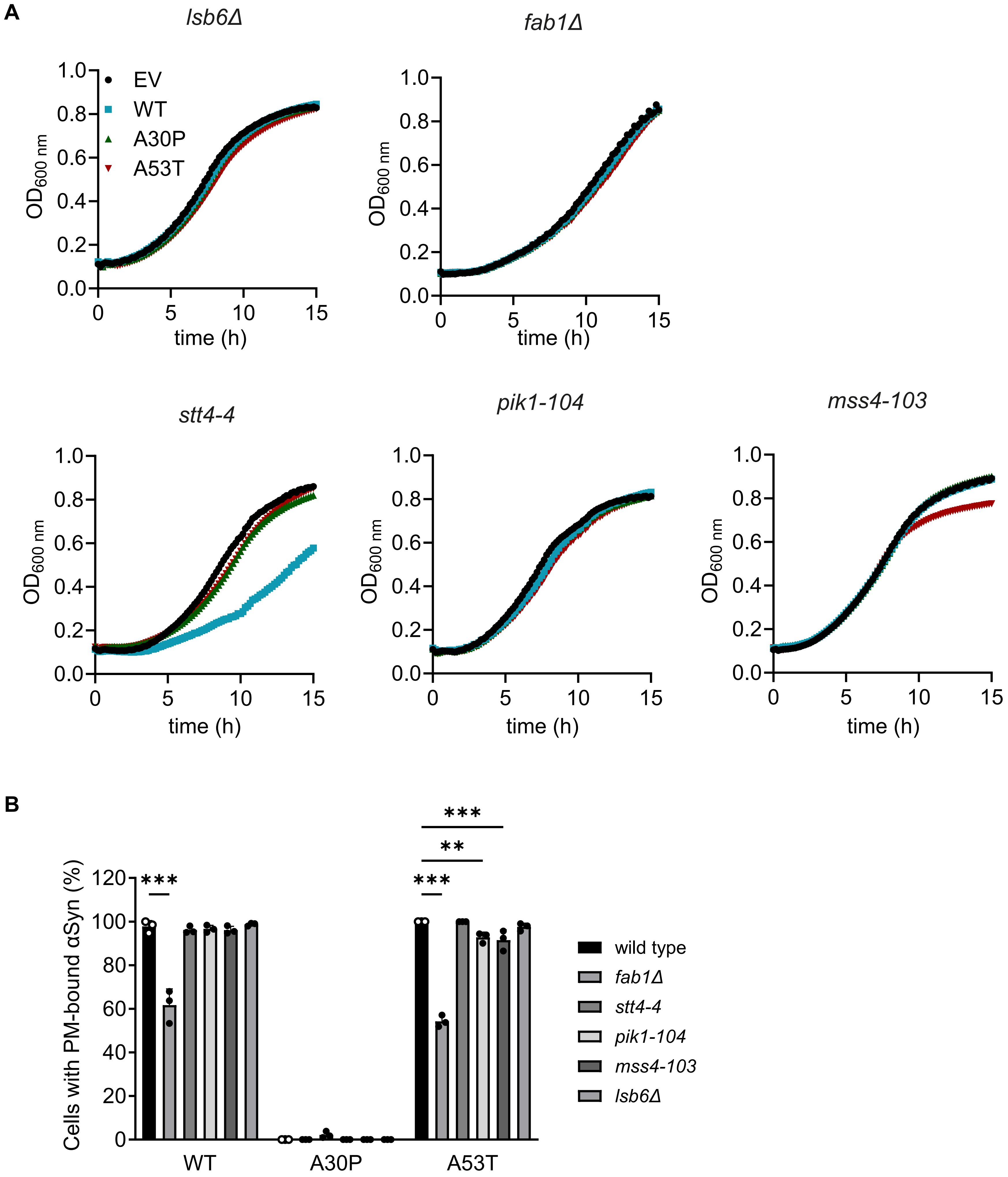

Figure S5: Expression of αSyn-GFP differentially affects PIP kinase deficient strains. A: Growth curves of kinase mutants *lsb6Δ*, *fab1Δ*, *stt4-4*, *pik1-104*, and *mss4-103* expressing WT, A30P, and A53T αSyn-GFP or the EV control from one-copy plasmids. Exclusively in the *stt4-4* strain, expression of WT αSyn-GFP induces growth inhibition. N = 3. **B:** Quantification of plasma membrane localization of WT, A30P, and A53T αSyn-GFP in the PIP-kinase deficient strains. Only in the *fab1Δ* strain, WT and A53T αSyn-GFP are substantially less localized at the PM. N = 3; values show mean + standard deviation. **$p < 0.01$; ***$p < 0.001$.

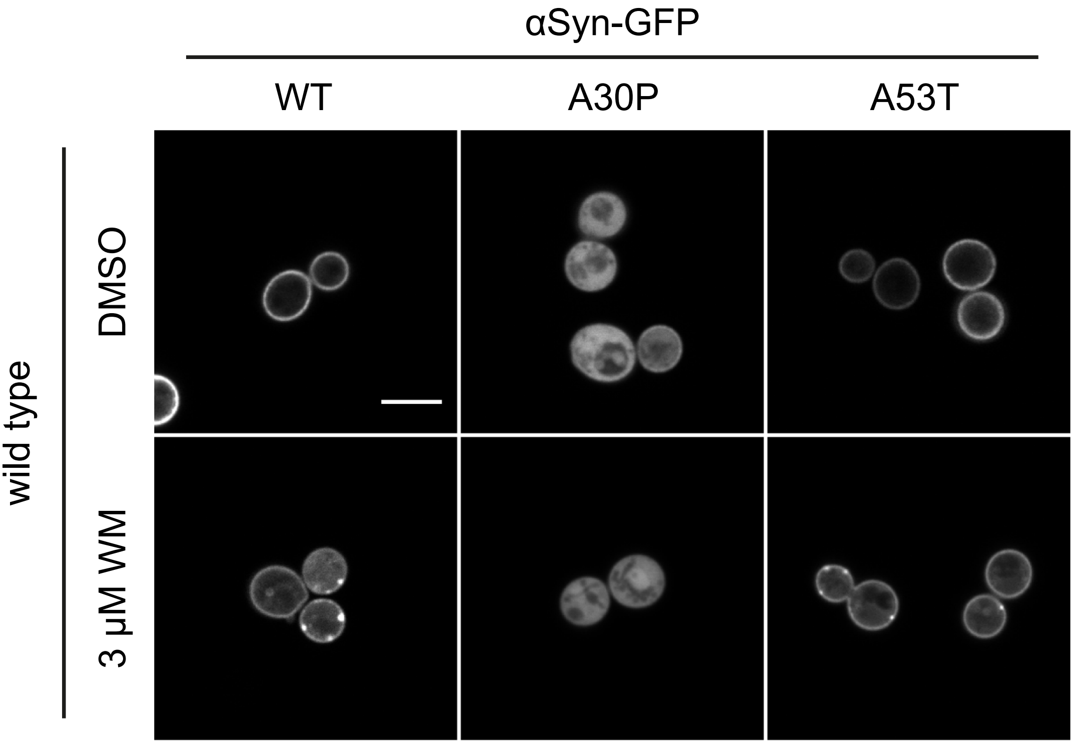

Figure S6: Wortmannin treatment mimics *stt4-4* phenotype. Treatment of wild type cells expressing WT, A30P, and A53T α Syn-GFP with 3 µM Wortmannin (WM) causes PM-localized accumulation of membrane-binding WT and A53T αSyn species, which is similar to the phenotype in the *stt4-4* strain. Scale bar: 5 µm.

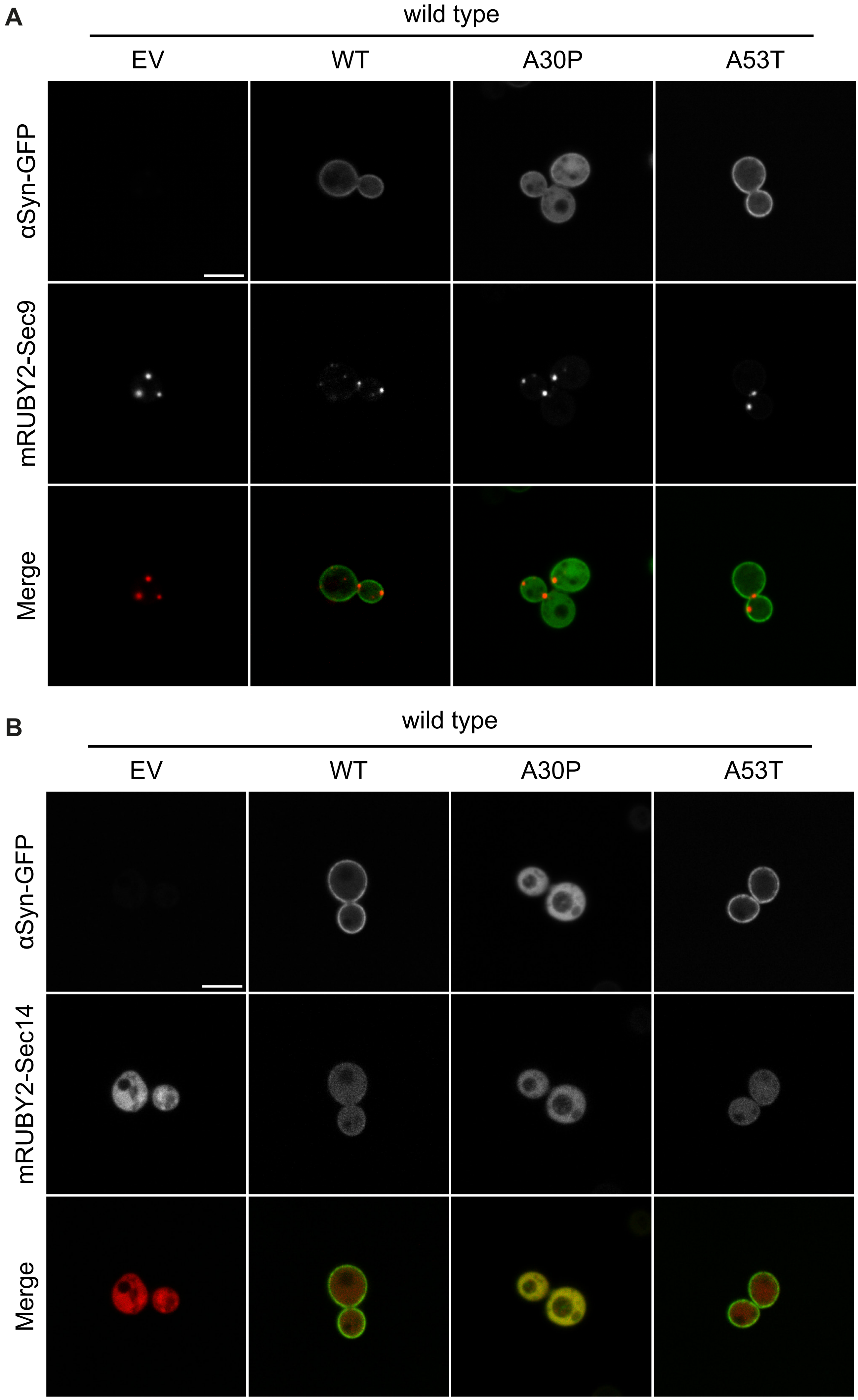

Figure S7: Overexpression of mRUBY2-Sec9 and mRUBY2-Sec14 does not affect αSyn-GFP localization. mRUBY2-Sec9 and mRUBY2-Sec14 were overexpressed in wild type cells expressing WT, A30P, and A53T αSyn-GFP. **A:** PM-bound t-SNARE protein mRUBY2-Sec9 does not affect αSyn-GFP localization. **B:** Cytoplasmic phospholipid transporter mRUBY2-Sec14 has also no effect on αSyn localization. Scale bar: 5 μm.
